# Psychiatric manifestations of Wilson's disease and treatment with electroconvulsive therapy

**DOI:** 10.4103/0019-5545.58898

**Published:** 2010

**Authors:** Manoj Kumar Sahoo, Ajit Avasthi, Madhusmita Sahoo, Manish Modi, Parthasarathy Biswas

**Affiliations:** Department of Psychiatry, Postgraduate Institute of Medical Education and Research (PGIMER), Chandigarh, India

**Keywords:** Electroconvulsive therapy, treatment, Wilson's disease

## Abstract

Wilson's disease is a rare genetic disorder involving the liver and brain, with onset frequently in adolescence. Psychiatric symptoms are often the first manifestation of the disease and can obscure the diagnosis. Although such patients are more commonly seen in neurological and hepatological settings, mental health professionals must keep in mind a high level of suspicion, once first presentations may be of psychiatric nature. We present the case of a 20-year-old female patient who initially presented with psychiatric symptoms. The neuropsychiatric manifestations and treatment of this patient with electroconvulsive therapy is presented.

## INTRODUCTION

In 1912, Dr. Kinnier Wilson published a description of 12 patients who presented with extrapyramidal motor disease and, on autopsy, demonstrated softening of the lenticular nucleus and cirrhosis of the liver.[1] He further noted that these patients exhibited “emotionalism,” and two of his 12 patients presented with schizophrenia-like psychoses. Across studies, up to one-fifth of patients with Wilson's disease (WD) present initially with psychiatric features in isolation, one-third present with predominating psychiatric features and two-thirds of patients with WD eventually develop psychiatric features.[2] The exact lifetime prevalence of psychiatric disorders is not known but is estimated to range between 30-100%.[3–5] Half of patients may undergo psychiatric hospitalization before WD is recognized. Psychiatric manifestations may precede neurological signs in the early stages of WD.[3] Incongruous behavior, irritability, depression, and cognitive impairment were the most common psychiatric symptoms among patients with WD.[5] Cognitive impairment is generally mild, occurring in less than 25% of patients.[4,6–8] The lifetime prevalence of persistent personality change ranges between 46-71%,[8,9] typically manifesting as irritability or aggression.[5,6,8,10,11] Schizophreniform disorders, catatonia, and hallucinations are no more common in WD than in the general population[4,6] but psychosis and catatonia occurred somewhat more commonly (8% each) in neurological WD.[9] Essentially, nothing is known about mania or its prevalence in WD. Major depression occurred in 27% in a prospective study of 45 subjects with WD.[6] Suicidal behavior occurs in 4-16% of patients with WD across studies.[3,6] As with mania, essentially nothing is known about anxiety disorders, substance abuse, or other psychiatric disorders in WD. Clinical improvement in WD with treatment is generally limited to the first five years of symptoms and the first two years of WD treatment.[3,12] Early recognition and treatment are therefore essential. Treatments include cupriuretic copper chelators (penicillamine and trientine). Dimercaprol a chelator was the earliest treatment for WD. Potassium disulfide, ascorbic acid, and vitamins E are, as yet, of unproven value. Controlled trials of psychotropic treatment, psychotherapy, and family therapy have not been carried out in WD.[13] It is anecdotally reported that patients with WD are predisposed to extrapyramidal side-effects with antipsychotics.[14] Electroconvulsive therapy (ECT) for delusional depression in a patient with WD led to a 3-min motor seizure followed by a switch to mania.[15] Further ECT treatment was interrupted by orthopedic surgery after a fall related to manic behavior. The authors pondered whether this case suggests greater sensitivity to ECT due to intracerebral copper deposition. However, there are a few case reports of successful ECT treatment for psychiatric symptoms in WD.[16] Beyond these rare anecdotes, little is known about treating psychiatric conditions in WD. The current case illustrates the various psychiatric manifestations of WD and treatment with a combination of atypical antipsychotics and ECT.

## CASE REPORT

A 20-year-old single female, with no family history of psychiatric illness was referred to Psychiatry Out Patient Department (OPD) from Neurology for management of acute psychiatric disturbances. Detailed exploration and review of neurology records revealed that in 1998 over a period of three to four months she developed decreased attention and concentration in studies, social withdrawal, drooling of saliva, slurring of speech, apathy, lack of interest in routine or pleasurable activities along with catatonic symptoms i.e. posturing, staring and mutism. She was diagnosed as WD, on the basis of serum ceruloplasmin-7.7 mg/dl (20-60), serum copper 60 μg/dl (80-155), 24 h urine copper 88 μg (10-30) and presence of Kayser-Fleischer ring on slit-lamp examination. She was started on Tab Pencillamine 500 mg/d and Zinc 150 mg/d and improved markedly except minimal drooling of saliva and slurring of speech. She remained well for the next four years with regular compliance to medications, and then stopped pencillamine. Subsequently she developed irritability, anger outbursts and violence, became socially disinhibited, tried to end her life, became suspicious of family members and reported visual hallucinations. During this time drooling of saliva, speech difficulty worsened and she also had one episode of generalized seizure. She was prescribed Tab Olanzapine 5-15 mg/d, along with Zinc acetate 150 mg/d and Tab Pencillamine 500 mg/d. She started showing improvement and recovered after three to four months with minimal drooling of saliva and difficulty in articulation of speech. She stopped medications again in November 2005. Subsequently, she was observed to be quiet, withdrawn and drooling of saliva from mouth worsened. She developed mutism, negativism, posturing, agitation, persecutory and referential ideas over one week and these symptoms continued with a fluctuating course. She did not improve with combination of Olanzapine, Zinc and pencillamine. From Psychiatry OPD along with above medications, tab Lorazepam was added up to 8 mg/d. She did not improve and was admitted to the psychiatry ward in January 2006. Mental status examination revealed negativism, rigidity and mutism. Neurological examination revealed rigidity of all four limbs, hypophonia and impaired saccades and pursuits. Serum ceruloplasmin-9.7 mg/dl (20-60) and serum copper-77 mg/dl (80-155) were low; 24 h urinary copper level was found within normal limits. Ultrasound abdomen revealed hepatosplenomegaly and Magnetic Resonance Imaging (MRI) brain revealed mild cerebral atrophy. Since no improvement was seen with medications, a course of eight ECTs was planned and administered. No post-ECT complications were observed. She improved completely and was discharged after three weeks. Medications were continued and she is maintaining well with regular follow- up and compliance with medications.

## DISCUSSION

Psychiatric manifestations may precede neurological signs in the early stages of WD. About 20% of them precede hepatic and neurological dysfunction.[2] This patient initially presented with social withdrawal, apathy followed by catatonic signs in the form of mutism, staring and posturing. Simultaneously she also developed drooling of saliva and dysarthria. She was diagnosed as a case of WD on the basis of biochemical abnormalities and presence of Kayser-Fleischer ring. Initial psychiatric manifestations were controlled with Tab Pencillamine and Zinc. Subsequently there were two relapses of psychiatric symptoms following stoppage of medications. Each relapse was characterized by violence, socially disinhibited behaviors, catatonic signs, persecutory and referential ideas and visual hallucinations. First relapse was controlled with combination of Tab Pencillamine, Zinc and, Olanzapine. Second relapse could not be controlled with the above combination of medicines. Hence, she was admitted to the psychiatry ward and administered ECT. This case illustrates various neuropsychiatric manifestations of WD, how these worsen and become difficult to treat with the subsequent relapses/exacerbations. The case also shows treatment with Olanzapine without much side-effects. Though patient developed mild extrapyramidal symptoms with Olanzapine, they improved on adding Trihexyphenydyl. With ECT patient did not develop prolonged seizure unlike the case reported earlier.[15] No other side-effects emerged during ECT. This case adds to the isolated report of treatment of psychosis with ECT in WD.[16] It can be concluded that ECT can be given in WD patients without much apprehension of complications, though systematic studies are needed to confirm the above findings.
